# Nothing personal, it’s the organization! Links between organizational culture, workplace bullying, and affective commitment

**DOI:** 10.3389/fpsyg.2024.1293610

**Published:** 2024-09-17

**Authors:** Eleanna Galanaki, Nancy Papalexandris, Irene Zografou, Nikolaos Pahos

**Affiliations:** ^1^Department of Marketing and Communication, HRM Laboratory, School of Business, Athens University of Economics and Business, Athens, Greece; ^2^Department of Values, Technology and Innovation, TU Delft, Delft, Netherlands

**Keywords:** workplace bullying, organizational culture, affective commitment, negative acts questionnaire, GLOBE project, SEM, repeated survey

## Abstract

Extensive attention in organizational research has been dedicated to workplace bullying, primarily focusing on its frequency and impact on both the victim and the bully, emphasizing interpersonal dynamics. This study extends current research by shifting the focus to the organizational level, examining the relationship between organizational culture and affective commitment, mediated by workplace bullying. Utilizing data from two surveys (*N* = 650 in 2012 and *N* = 553 in 2017), the study reveals that dimensions of organizational culture, such as assertiveness, performance orientation, and ingroup collectivism significantly influence work-related workplace bullying. Performance orientation and assertiveness are positively associated with increased bullying, whereas ingroup collectivism serves as a deterrent. In turn, work-related bullying negatively impacts affective commitment, while a culture characterized by high ingroup collectivism not only links negatively with bullying but also links positively with affective commitment. This work is one of the first studies to investigate the interplay among several dimensions of organizational culture, workplace bullying, and affective commitment, underscoring the importance of supportive organizational cultures in fostering healthy work environments.

## 1 Introduction

Bullying as a phenomenon occurs when individuals experience consistent and prolonged negative treatment or psychological attack from one or more individuals, where they struggle to defend themselves or to escape (Ahmad et al., 2021). Bullying is a significant source of stress in the workplace, causing significant distress to individuals (Hauge et al., 2010). The term of workplace bullying emerged in the early 1990s to represent a common yet serious phenomenon (Galanaki and Papalexandris, 2013). During that period, it became evident that employees experienced significant stress due to persistent and repeated intimidating and demeaning behaviors they encountered, which they found challenging to confront. Today, bullying has become a prevalent social problem within contemporary workplaces, affecting 11–18% of the global workforce (Ahmad et al., 2021). The Workplace Bullying Institute computed the percentage of working US citizens affected by bullying at 49% (30% as victims and 19% as witnesses) (Namie, 2021), while empirical evidence from India has estimated it at 46% (Gupta et al., 2017).

Specifically in Greece, a recent survey covering economically active Greek citizens from all geographic areas has revealed that 85% of respondents believe that workplace bullying is so widespread that it should be considered a crucial social problem (Κapa Research SA, 2021). Specifically, 38% had experienced bullying in their workplace, 79% witnessed oral abuse, 65% intimidation and threats, and 54% encountered false rumors or negative comments. However, 48% of employers reported lack of prevention measures in their company. Therefore, bullying prevention measures have intensified globally (Escartín, 2016). In Greece, recent legislation integrates the ILO convention against violence and harassment at work. Proposed prevention measures include top management commitment, identification of possible risk factors, designing a prevention strategy and policies which will advocate zero tolerance to bullying, sensitivity training of managers and employees, and assigning a person responsible for communicating policies, organizing training, receiving complaints, and examining every instance of bullying reported.

Previous research in the field of organizational studies, has associated workplace bullying with organizational concepts such as organizational culture (e.g., Tambur and Vadi 2012). Organizational culture varies by sector and cultural context in which the organization operates (Ahmad et al., 2021; Naseer et al., 2018). For instance, workplace bullying is more prevalent in the labor-intensive sectors such as hospitality (Hayat and Afshari, 2021; Srivastava and Agarwal, 2020), education, health, and sports sectors (Allen et al., 2015; Vveinhardt and Fominiene, 2020). In a recent volume covering bullying in different sectors and occupations (D'Cruz et al., 2021), it is evident that several organizational factors are related to workplace bullying and that bullying is more prevalent in some sectors of the economy and in specific professional roles. For example, security forces (e.g., police, fire brigade, army), care and service professions (nurses and social workers, hospitality, public service), education establishments (schools and universities) and non-standard employment arrangements (such as dirty work and precarious jobs) have been studied extensively for the occurrence and antecedents of workplace bullying (e.g., D'Cruz et al., 2021). Taking organizational features into consideration, Gamian-Wilk and Madeja-Bien (2018) highlighted that organizational culture May enhance or hinder the occurrence of workplace bullying, possibly regardless of the sector in which the organization operates or the types of jobs executed. Moreover, within the framework of cultural distinctions, a global study conducted on six continents examined the acceptability of workplace bullying and found that individuals in countries characterized by a significant power distance are more inclined to accept workplace bullying (Ahmad et al., 2021; Power et al., 2013). Recently, emphasis has also been given to national cross-cultural differences and similarities in perceptions of bullying (Salin et al., 2019).

However, overall, the dynamics between workplace bullying and organizational culture, need further exploration. Given the unique organizational culture manifested in any workplace, often referred to as the “way we do things around here” or “a set of common beliefs, norms, and values” (Schein, 1992), this study aims to explore the potential link or causal relationship between organizational culture and workplace bullying. Also, while previous studies have identified negative consequences of bullying in terms of employee behaviors, such as organizational commitment, the explanation of such relationship remains underexplored (Parzefall and Salin, 2010). Therefore, the current study seeks to examine how organizational culture relates to the occurrence of workplace bullying and affective commitment. Understanding the factors underlying bullying and reinforcing cultural characteristics that act as obstacles, can help companies face this phenomenon, which lowers productivity and hurts employee morale and engagement. By identifying cultural dimensions related to bullying, violence, harassment or ostracism in the workplace, policies can be better designed, training better planned, and values and norms redefined with employee involvement. Values and personal characteristics which do not favor bullying can also serve as guidelines by Human Resource Management (HRM) practitioners in hiring Decisions.

To comprehend the influence of organizational culture on workplace bullying within an unstable context, and its subsequent impact on employee attitudes, such as affective commitment, this study is conducted in Greece, a country that has recently gone through a deep macro-level recession, creating opportunities for examining the relations under study in a changing and volatile environment. Specifically, this research was run in two research rounds, in 2012 when the recession was still on the rise and again in 2017 when economic recovery was underway (Galanaki, 2020a,b). This paper examines three key variables, i.e., organizational culture, workplace bullying, and affective organizational commitment, underpinning the following inquiries:

How are the dimensions of culture associated with the occurrence of workplace bullying?
How does workplace bullying link with overall organizational commitment?
How does organization culture relate with affective commitment, through the effects of workplace bullying?

**Figure 1:**
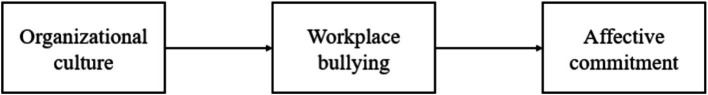
Conceptual model.

The following figure illustrates the connections to be tested between the three key variables (see Figure 1).

## 2 Literature review

### 2.1 Workplace bullying

The body of literature concerning workplace bullying can be categorized into two overarching groups of studies.

#### 2.1.1 Studies centered on the measurement and evaluation of the bullying phenomenon

While workplace bullying has been a subject of scientific inquiry for many years, a significant challenge in the realm of workplace literature remains the assessment of its occurrence. Recent papers have been increasingly dedicated to addressing the question of how workplace bullying should be measured, as in the work of Galanaki and Papalexandris (2013). Previous literature has also distinguished between work-related and person-related bullying behaviors (Einarsen, 1999). Specifically, behaviors such as “slander, social isolation and insinuation about someone’s mental health May be seen as examples of person-related bullying, whilst giving a person too many, too few or too simple tasks, or persistently criticizing a person or their work, May be associated with work-related bullying” (Einarsen et al., 2009, p. 26).

In addition, Nielsen et al. (2009) conducted a comprehensive review of international studies that reported the prevalence of workplace bullying. The authors found that the percentage of bullying occurrences varied widely, ranging from 1 to 55%. This variation was attributed to the measurement methods employed and the geographical location of these studies. Therefore, despite variations in the exact reported occurrence levels, contingent on the study and research setting, it is evident that workplace bullying is a reality, and its occurrence has been consistently recorded in previous research.

#### 2.1.2 Studies dedicated to investigating the consequences of the bullying phenomenon

Among the reported effects of workplace bullying are stress, anxiety, irritability, depression, mood swings, feelings of helplessness, lowered self-esteem, despair, burnout, alienation, social isolation and maladjustment, physical symptoms (Kerse and Babadag, 2019; Lutgen-Sandvik, 2008), and lowered job satisfaction (Arenas et al., 2015; Plimmer et al., 2022; Sprigg et al., 2019). Many researchers have directed their attention toward examining the long-term effects of bullying at work, with a notable emphasis on conditions such as post-traumatic stress disorder (PTSD) (Mikkelsen and Einarsen, 2002; Nielsen et al., 2015). In addition to its direct effects, bullying also exerts indirect influences on long-term behaviors. One of the most distinctive characteristics, is the victim’s tendency towards counter-aggression (Chenevert et al., 2022; Escartín et al., 2021).

An alternative way to approach workplace bullying is from the organizational standpoint. The effects of bullying extend beyond its victim (s). Considerable research has explored the organizational or group-level effects, including the impact on the overall working environment (Finstad et al., 2019), performance and productivity (Merilainen et al., 2019; Wu et al., 2020), increased absenteeism (Lene, 2023), or the intention of employees to leave (Ahmad and Kaleem, 2020; Djurkovic et al., 2008). Conversely, organizations bear the dual responsibility of contributing to the occurrence of bullying (Plimmer et al., 2022) and for reducing both the frequency of this phenomenon and the extent of harm it causes (Bulutlar and Öz, 2009; Ribeiro et al., 2022).

### 2.2 The role of organizational culture

Organizational culture is regarded by business and organizational scientists as both a pivotal factor for improvement and a potential barrier when attempting to modify negative behaviors. In simple terms, culture signifies “the way we do things around here,” and it can both foster a sense of belonging among group members and create a divide between them and non-members. Therefore, organizational culture May either facilitate or hinder workplace bullying. However, a culture of respect is typically suggested as a means to reduce the likelihood of workplace bullying occurrences (Robotham and Cortina, 2021). This highlights the need to address hostile and unethical work environments and potential autocratic leadership behaviors (Escartín et al., 2021; Hoel et al., 2009). However, there has not been any substantial research linking established dimensions of organizational culture with the occurrence of workplace bullying.

One of the most recognized studies on organizational culture is the GLOBE project, led by the late Robert House. This methodological approach draws from Hofstede’s work and represents one of the most prominent international initiatives to study organizational culture (House et al., 2004). The GLOBE methodology assesses nine dimensions of organizational culture, which are presented below, along with the way in which they May link with bullying occurrence and affective commitment:

- Uncertainty avoidance: Refers to the practices adopted to minimize or avoid the uncertainty that exists among members of the organization. Potentially in organizations where uncertainty avoidance is high, administrative mechanisms that diminish the risk of unacceptably aggressive or demeaning behaviors are more probable to exist as a way to reduce the risk of litigation and bad reputation, therefore bullying May be more restrained. Also, in such organizations, individuals generally experience lower levels of uncertainty, which May make them feel more at ease in their role as members of the organization, therefore fostering their affective organizational commitment.
- Future orientation: Indicates the extent to which individuals in organizations engage in future-oriented behaviors, such as planning, investing in the future, and delaying gratification. Future orientation refers to the value of the future over present benefits. In organizations where future orientation is high, the preservation of existing relationships and resources (including human capital) of the organization is important. Therefore, human relations are valued in a way that fosters commitment to the organization and encourages precautions to avoid bullying phenomena.
- Power distance: Relates to the degree of centralization and the gap in power between different hierarchical levels within an organization. Power distance has already been related to the experience and interpretation of workplace bullying and affective commitment (Nguyen et al., 2024)
- Institutional collectivism: Measures the extent to which an organization values cooperative over individualistic behavior. Collectivism has already been linked to affective organizational commitment (Galanaki et al., 2020). Moreover, the value of cooperation over individualistic behavior that collectivism upholds May hinder bullying behavior, as intimidating behaviors go against the (Galanaki et al., 2020) benefits of the collective.
- Humane orientation: Reflects the support of human beings, including qualities such as generosity, concern, and friendliness, within an organization. These values are inherently opposed to bullying behaviors (Power et al., 2013) and nurture the development of feelings of belongingness and identity, therefore supporting affective commitment to the organization.
- Performance orientation: Represents the degree to which an organization encourages and rewards group members for performance improvement and excellence. Research has already established that cultures with high performance orientation find bullying to be more acceptable (Power et al., 2013) and that customer orientation links with affective commitment (Zang et al., 2021).
- Ingroup collectivism or family collectivism: Pertains to the emphasis on family/internal group bonds within an organization, highlighting the strength of the family/friend connections. As with institutional collectivism, ingroup collectivism has been found to act as a buffer to workplace bullying (Karatuna et al., 2020) and to link with affective organizational commitment (Galanaki et al., 2020).
- Gender egalitarianism: Refers to the extent to which the organization supports the equal treatment between the two genders. Gender egalitarianism May hinder workplace bullying in an indirect way, as bullying itself appears to be a generally gendered phenomenon (Escartín et al., 2011; Rosander et al., 2020). Also, affective commitment appears to be linked to gender (Shin et al., 2020).
- Assertiveness: Measures the degree to which individuals are encouraged to be dominant and assertive within an organization. Research is inconclusive towards whether assertiveness hinders or encourages workplace bullying. At the individual level, being more assertive minimizes the risk of becoming a target to bullying, but when the environment (including supervisors and coworkers) is very assertive, this May lead to bullying experience by weaker individuals (Fang et al., 2020; Gamian-Wilk et al., 2022). There is no existing evidence on how assertiveness May be linked with workplace bullying.

### 2.3 Effects on affective organizational commitment

An employee attitude that has traditionally received significant attention when examining its connection to individual and organizational effectiveness and outcomes is organizational commitment (Jehanzeb and Mohanty, 2018). However, despite the extensive research on this attitudinal trait and its relationship with multiple effectiveness indicators, its connection with workplace bullying has not been extensively explored or thoroughly investigated. In fact, workplace bullying effects mostly focus on behaviors, such as performance and productivity (Merilainen et al., 2019), counter-aggression, absenteeism (Lene, 2023), or sickness absenteeism (Nielsen et al., 2019), rather than on attitudes. With the exception of one recent study that analyses the impact of witnessing workplace bullying on employee well-being and attitudes (Salin and Notelaers, 2020), there is only a handful of studies that specifically concentrate on the intention to leave (Ahmad and Kaleem, 2020; Djurkovic et al., 2008; Houshmand et al., 2012), which it is important to note, is not purely an attitude, but rather an intention related to a particular behavior.

From the three categories of organizational commitment, i.e., affective, normative, and continuance (Nandan et al., 2018), we choose to focus on affective commitment that reflects an emotional connection to, a sense of identification with, and active engagement in the organization (Meyer and Allen, 1991). In this regard, affective commitment is considered high when employees experience a sense of attachment and belonging or a feeling that they are “part of the family” in their organizations (Colquitt et al., 2014). Affective commitment has been described as “the core essence of organizational commitment” (Mercurio, 2015) and it is the dimension of organizational commitment that has been linked most with positive organizational behaviors and outcomes (Lam and Liu, 2014).

There is research evidence documenting that workplace bullying, being a negative phenomenon to both victims and observers, links negatively to affective commitment (McCormack et al., 2006; Steele et al., 2020). This makes sense because feeling as “a member of a family” and developing affect for an employer is difficult when the employer does not protect you from experiencing bullying. On the other hand, affective commitment May act as a buffer to negative effects of workplace bullying (Cooper-Thomas et al., 2013; Liu et al., 2020).

**Figure 2:**
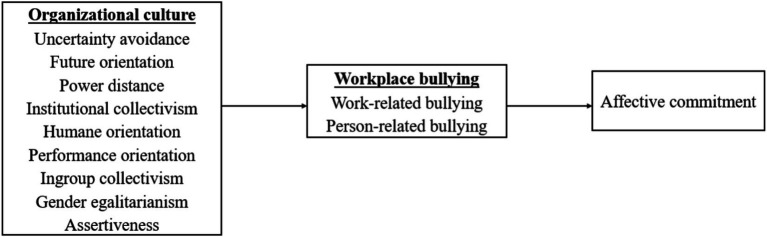
Expanded conceptual model.

Figure 2 further elaborates on the conceptual framework, illustrating the dimensions of organizational culture and workplace bullying that are measured in this study.

## 3 Research methodology

### 3.1 Data collection

A survey involving 650 white-collar workers was conducted in 2012. In 2017 the survey was repeated with the same research instrument and participation of 553 *other* respondents, in agreement to the repeated survey methodology (Firebaugh, 1997), which allows “a shift of focus, from an investigation of individual-level microprocesses to one of aggregate-level macroprocesses” and offers the “opportunity to analyze changes in society” (*ibid.*: v). We chose this method to confirm that the initial measurements were correct and to control for the effects of the prolonged recession in Greece. Convenience sampling was employed: responses from 4 to 10 employees with a minimum of 2-years of work experience at each visited organization were collected. Employees were requested to fill out a questionnaire.

A structured questionnaire was used. To ensure accurate translation into Greek, the direct translation—back-translation method (Brislin, 1970) was employed. To determine the study’s sample, the iMentor database, an online platform provided by Infobank-Hellastat (), containing contact details for over 100,000 private and public Greek companies was leveraged. Three hundred companies were randomly selected from this database. Contact with these firms was established via telephone or email, seeking permission to collect data from their employees and offering feedback on their organizational culture in return. To streamline the data-collection process, students were trained as research assistants. These assistants distributed questionnaires to employees who had a minimum of 2 years of experience within the current organizations, ensuring that respondents possessed sufficient experience to evaluate the culture of their organizations accurately. Two weeks later, the research assistants collected the completed questionnaires directly from the respondents, who sealed them in anonymous envelopes. The data-collection process resulted in 650 (of 945) individual responses in the first research round and 553 (of 1,018) individual responses in the second research round. In both rounds, eleven and sixteen responses were excluded, respectively, due to incomplete responses on the research instrument.

### 3.2 Sample

The final total sample in the two research rounds comprised 136 organizations, with 66.3% being private organizations and 33.7% being public organizations. On average, there were approximately eight respondents per organization. The completed questionnaires were provided by individuals with an average work experience of 15.78 years, representing a diverse range of functions, departments, and vocational specializations, including management (38%), sales (18%), engineering (5.1%), HRM (4.8%), procurement (2.7%), and support services (8.3%). Most of the respondents were female, accounting for 53.6% of the sample. The respondents’ average age was 39.87 years, with a standard deviation of 9.33, and age ranged from 19 to 70 years. Table 1 provides an overview of the collected sample, i.e., the profile of the employees answering the questionnaire in each research round.

**Table 1:** Descriptive statistics (2012, 2017).

	2012				2017			
	Mean	Min	Max	Std. D	Mean	Min	Max	Std. D
Age	38.87	20	65	8.81	39.87	19	70	9.33
Years in education	16	1	28	3.05	16.13	4	40	2.86
Working Experience	14.87	1	36	8.92	15.80	1	50	9.30
Working Experience with current Employer	9.76	0	36	8.53	10.42	2	50	8.76
Number of subordinates	3.98	0	140	12.83	4.06	0	150	11.55
Organizational levels to the top	3.04	0	13	2.25	2.32	0	8	1.44
Organizational levels to the bottom	1.29	0	7	1.55	1.08	0	7	1.24

		2012	2017
Traits of respondent	Male	43%	50%
	Female	57%	50%
	Married	55%	58%
	With children	48%	58%
Department	Management	23%	17%
	Economics	26%	35%
	Engineering, production	4%	9%
	Mathematics/Physics/	3%	3%
	Informatics	9%	8%
	Classics/Languages	11%	12%
	Other	17%	15%

### 3.3 Measurement and scales

#### 3.3.1 Organizational culture: nine dimensions

Organizational culture was measured and evaluated using the GLOBE^1^ questionnaire on Organizational culture (GLOBE phase 2, 2004 study). This instrument has gained widespread recognition for assessing 9 major cultural dimensions within organizations and is considered one of the most reliable scales for measuring organizational culture worldwide (House et al., 2004). The questionnaire employs a 7-point Likert-type scale to assess 9 cultural dimensions via 32 questions, providing insight into the existing organizational cultures and value systems.

The nine dimensions of organizational culture encompassed by the questionnaire are presented in Table 2.

**Table 2:** Measurement items of organizational culture.

Organizational culture dimension	Scale	Indicative items
Uncertainty avoidance	3-item scale	In this organization, orderliness and consistency are stressed, even at the expense of experimentation and innovation (strongly agree: 1; strongly disagree: 7).
Future orientation	3-item scale	The way to be successful in this organization is to: (plan ahead:1; take events as they occur: 7)
Power distance	3-item scale	In this organization, subordinates are expected to: (obey their boss without question: 1; question their boss when in disagreement: 7)
Institutional collectivism	3-item scale	In this organization, managers encourage group loyalty even if individual goals suffer. (strongly agree: 1; strongly disagree: 7).
Humane orientation	4-item scale	In this organization, people are generally: (very concerned about others: 1; not at all concerned about others:7)
Performance orientation	4-item scale	In this organization, employees are encouraged to strive for continuously improved performance. (strongly agree: 1; strongly disagree: 7).
Ingroup collectivism	5-item scale	In this organization, group managers take pride in the individual accomplishments of group members. (strongly agree: 1; strongly disagree: 7).
Gender egalitarianism	3-item scale	In this organization, men are encouraged to participate in professional development activities more than women. (strongly agree: 1; strongly disagree: 7).
Assertiveness	4-item scale	In this organization, people are generally (dominant: 1; non-dominant: 7).

#### 3.3.2 Workplace bullying

The occurrence of workplace bullying was assessed with the Negative Acts Questionnaire (NAQ), which includes 21 categories of Negative Acts, that May occur in the working environment, such as intimidation, ongoing critique or criticism of work and efforts, spreading rumors, and making false allegations, among others. The NAQ is widely recognized and accepted as a reliable instrument for measuring workplace bullying (Einarsen et al., 2009). Participants were presented with 21 statements describing various negative acts and asked to rate their frequency on a scale from 1 = never, 2 = yes, occasionally, 3 = yes, at least once a month, 4 = yes, at least once a week, 5 = yes, every day. Importantly, these questions were asked in terms of their entire working career rather than a specific timeframe. Then, following the Einarsen et al. (2009) methodology, we extracted two factors, namely work-related bullying and person-related bullying, which were extracted and confirmed with CFA in STATA. During the reliability analysis, some questions from the original factors proposed by Einarsen et al. (2009) were removed due to high cross-loadings between the two measures, work-related bullying and person-related bullying.^2^ Work-related bullying consisted of 3 items, namely “Being given tasks with unreasonable deadlines,” “Pressure not to claim something to which by right you are entitled (e.g., sick leave, holiday entitlement, travel expenses),” and “Being exposed to an unmanageable workload.” Person-related bullying consisted of 7 items, including for example, “Being humiliated or ridiculed in connection with your work” and “Being ignored or facing a hostile reaction when you approach.”

#### 3.3.3 Affective organizational commitment

Affective Organizational Commitment was measured with the Allen and Meyer (1990) commitment scale. This instrument is widely recognized and accepted as one of the most well-established tools for measuring organizational commitment (Nandan et al., 2018). The questionnaire employs a 5-point Likert-type scale with 8 items to measure affective organizational commitment. Indicative items are “I really feel as if this organization’s problems are my own” and “I do not feel like “part of the family” at my organization (reverse scored).

### 3.4 Confirmatory factor analysis

As discussed above, in this study very popular and highly cited scales for measuring all variables were adopted. As a result, a Confirmatory Factor Analysis (CFA) for the organizational culture, workplace bullying and affective commitment items was conducted, employing STATA 16 software for the analysis. It must be noted that for organizational commitment and workplace bullying, which are higher order constructs, we compared alternative models in order to show that the first-order constructs should be treated as distinct variables.

Table 3 presents the fit indices for all constructs. Maximum likelihood estimation was used to calculate the loadings of the variables, and a chi-square test was performed to assess the fit of the models. All goodness-of-fit indices were also considered, including the comparative fit index, the Tucker Lewis index, the standardized root mean square residual and the root-mean-square error of approximation. The models’ fit statistics fall within recommended standards (Hu and Bentler, 1999), indicating a good model fit. Results also show that the 9-factor model for organizational culture and the 2-factor model for workplace bullying had a better model fit than the respective 1-factor models.

**Table 3:** CFA goodness of fit indices for organizational culture dimensions, workplace bullying dimensions and affective commitment (both rounds).

Construct	Χ^2^	CFI	TLI	SRMR	RMSEA	CD
Organizational culture (9-factor model)	1862.35	0.90	0.89	0.06	0.05	1.00
Organizational culture (1-factor model)	3751.71	0.78	0.76	0.06	0.08	0.95
Workplace bullying (2-factor model)	206.85	0.97	0.96	0.04	0.07	0.97
Workplace bullying (1-factor model)	498.16	0.92	0.89	0.06	0.11	0.92
Affective commitment (1-factor model)	115.07	0.97	0.96	0.03	0.06	0.87

Subsequently, the internal reliabilities of the scales were examined and indicated acceptable Cronbach’s alpha values for all variables, ranging from 0.60 to 0.85, except for ‘Power distance’ and ‘Gender egalitarianism’ cultural dimensions (0.438 and 0.227 respectively). However, the two variables were accepted because the CFA indicated acceptable fit indices for the dimensions of organizational culture.

## 4 Findings

### 4.1 Description of the answers given

To present the findings regarding each of the primary variables under examination, Table 4 displays the descriptive statistics for each of the 8 Organizational Culture dimensions, workplace bullying occurrence and organizational commitment both for 2012 and 2017.

**Table 4:** Descriptive statistics for organizational culture, affective organizational commitment, and workplace bullying (2012, 2017).

Culture dimensions	2012		2017	
	Mean	Std. deviation	Mean	Std. deviation
Organizational culture				
Uncertainty avoidance	4.23	1.55	4.55	1.48
Future orientation	3.99	1.62	4.21	1.68
Power distance	3.76	1.31	3.58	1.19
Institutional collectivism	3.91	1.33	4.20	1.21
Humane orientation	4.42	1.10	4.60	1.13
Performance orientation	3.74	1.27	3.92	1.27
Ingroup collectivism	4.43	1.15	4.78	1.16
Gender egalitarianism	4.32	0.80	4.31	0.80
Assertiveness	3.57	1.02	3.45	1.04
Organizational commitment				
Affective Commitment (1–5)	3.26	0.78	3.41	0.75
Workplace bullying				
Person-related workplace bullying	1.61	0.72	1.69	0.76
Work-related workplace bullying	2.90	1.09	3.14	1.61

As mentioned above, a reason for running the research as a repeated survey with two research rounds was that we wanted to control for exogenous to our research aims effects, in this case the external environment, i.e., the economic condition of the country, being in a deep recession in 2012, and recovering from the recession in 2017. As appears in Table 4, almost all the variables are quite similar between the two research rounds. A test of difference of means was conducted between the two research rounds and the H_0_ for difference was rejected for all variables of the sample (*p* < 0.05). Only person-related bullying appears higher in 2017 than in 2012, and this difference is statistically significant (*t* = 2.8389, *p* = 0.00). Possibly this difference can be explained by the fact that the more beneficial macroeconomic environment in 2017 made respondents more confident and they felt safe to report incidents they perceived as personal attacks.

### 4.2 Relationships among the collected responses

To explore the connections we aimed to investigate, a correlation analysis encompassing all the organizational culture dimensions, workplace bullying and affective commitment was first conducted. The analysis was run first for each research round separately and then for the combined sample from both research rounds. No difference was observed in intercorrelations across research rounds. The results of the analysis for the combined sample including observations from both research rounds are presented in Table 5.

**Table 5:** Correlations matrix of all latent variables in the model (combined sample for research rounds 2012 and 2017).

	Variable	Cronbach’s a	Mean	Std. dev.	1	2	3	4	5	6	7	8	9	10	11	12
1	Uncertainty Avoidance	0.61	4.38	0.68	1.00											
2	Future Orientation	0.71	4.02	0.86	0.64	1.00										
3	Power distance	0.44	3.68	0.63	−0.44	−0.65	1.00									
4	Institutional collectivism	0.65	4.05	0.74	0.62	0.75	−0.73	1.00								
5	Humane orientation	0.81	4.50	0.68	0.57	0.65	−0.72	0.74	1.00							
6	Performance orientation	0.76	3.83	0.87	0.63	0.81	−0.57	0.75	0.67	1.00						
7	Ingroup collectivism	0.82	4.60	0.74	0.74	0.77	−0.68	0.77	0.77	0.82	1.00					
8	Gender egalitarianism	0.33	4.31	0.52	0.24	−0.05	0.08	0.08	0.03	0.17	0.18	1.00				
9	Assertiveness	0.66	3.52	0.55	−0.25	−0.29	0.65	−0.45	−0.66	−0.20	−0.37	0.19	1.00			
10	Work-related bullying	0.73	0.00	0.54	−0.16	−0.12	0.20	−0.16	−0.21	−0.07	−0.19	−0.06	0.22	1.00		
11	Person-related bullying	0.91	0.00	0.45	−0.09	−0.06	0.14	−0.10	−0.14	−0.04	−0.12	−0.07	0.17	0.77	1.00	
12	Affective commitment	0.83	3.33	0.78	0.34	0.30	−0.26	0.30	0.29	0.29	0.41	0.14	−0.13	−0.22	−0.12	1.00

As a next step, we performed a Structural Equation Modeling (SEM) with STATA 16, to further explain the underlying mechanism between organizational culture, workplace bullying and affective commitment. Specifically, the model was run in SEM in two groups, one for 2012 and one for 2017. Table 6 illustrates the results of the SEM analysis. Multi-level SEM was also run (with generalized SEM, STATA function GSEM) to account for fixed effects per company and this further, unreported analysis bared similar results to those reported in Table 4. We sticked with reporting results per year of study by groups in regular SEM, as the aim of the present paper was to ratify robustness of the model across research rounds, not companies.

**Table 6:** Results of SEM analysis (2012, 2017).

			Std. Coef.			Std. Coef.		
			2012		Std. Err.	2017		Std. Err.
*Work_related < −*								
Uncertainty_avoidance			−0.02		0.06	−0.13	***	0.07
Future_orientation			−0.04		0.08	−0.1		0.09
Power_distance			0.03		0.07	0.03		0.08
Institutional_collectivism			−0.03		0.07	0.03		0.08
Humane_Orientation			−0.05		0.08	−0.01		0.09
Performance_Orientation			0.30	***	0.09	0.25	***	0.08
Ingroup_Coll			−0.21	***	0.09	−0.19	**	0.1
Gender_Egalitarianism			−0.04		0.05	−0.15	***	0.05
Assertiveness			0.12	***	0.06	0.16	***	0.08
_cons			0.16		0.86	1.42		0.97
*Person_related < −*								
Uncertainty_avoidance			0		0.06	−0.08		0.07
Future_orientation			0.01		0.08	−0.02		0.09
Power_distance			0.11	**	0.07	−0.01		0.09
Institutional_collectivism			0.02		0.07	0.03		0.08
Humane_orientation			0.02		0.08	0.04		0.1
Performance_orientation			0.17	**	0.09	0.12		0.08
Ingroup_Coll			−0.13		0.09	−0.16		0.1
Gender_egalitarianism			−0.04		0.05	−0.17	***	0.05
Assertiveness			0.11	**	0.06	0.22	***	0.08
_cons			−1.38		0.87	0.87		0.99
Affective_commitment <−								
Work_related			−0.17	***	0.05	−0.27	***	0.07
Person_related			0.06		0.05	0.12	**	0.07
Uncertainty_avoidance			0.12	***	0.06	−0.07		0.06
Future_orientation			0.17	***	0.07	−0.05		0.08
Power_distance			−0.06		0.06	−0.02		0.08
Institutional_collectivism			−0.06		0.07	0.1		0.08
Humane_orientation			−0.05		0.07	0.03		0.09
Performance_orientation			−0.11		0.09	−0.2	***	0.08
Ingroup_Coll			0.25	***	0.08	0.57	***	0.09
Gender_egalitarianism			0.11	***	0.04	−0.03		0.04
Assertiveness			0.01		0.05	0.13		0.08
_cons			1.56		0.81	1.41		0.93

***p < 0.05, **p < 0.10.

Regarding the effects of organizational culture on workplace bullying, our results reveal generally similar effects for 2012 and 2017, especially in relation to work-related workplace bullying. In particular, performance orientation (*b* = 0.25, *p* < 0.05 for 2017; *b* = 0.30, *p* < 0.05 for 2012), ingroup collectivism (*b* = −0.19, *p* < 0.10 for 2017; *b* = −0.21, *p* < 0.05 for 2012), and assertiveness (*b* = 0.16, *p* < 0.05 for 2017; *b* = 0.12, *p* < 0.05 for 2012), had a significant impact on work-related workplace bullying in both time periods. Assertiveness was also found to relate significantly with person-related bullying for 2012 and 2017 (*b* = 0.11, *p* < 0.05; *b* = 0.22, *p* < 0.05).

Regarding the effects of workplace bullying on affective commitment, work-related bullying had a negative and significant effect on affective commitment in both time periods (*b* = −0.27, *p* < 0.05 for 2017; *b* = −0.17, *p* < 0.05 for 2012).

Overall, our findings suggest that the relationship between organizational culture and affective commitment, through the effects of workplace bullying (mediation), is relevant only for the dimension of work-related bullying. Specifically, there is an indirect relationship between organizational culture, work-related bullying, and affective commitment, only for the dimensions of performance orientation, ingroup collectivism and assertiveness. At the same time, ingroup collectivism was significantly related with affective commitment (*b* = 0.57, *p* < 0.05 for 2017; *b* = 0.25, *p* < 0.05 for 2012), implying a partial mediating mechanism between this cultural dimension, work-related bullying, and affective commitment.

## 5 Discussion

This section focuses on how the analyses address the original research questions. We will emphasize findings that were consistent across the two research rounds and briefly outline the key differences observed.

Among the relations examined, the first research question that requires attention is the fact that certain dimensions of organizational culture were significantly related to workplace bullying. The most telling case is that of the assertiveness cultural dimension, which expresses the degree to which individuals are encouraged to be dominant and assertive within an organization. Assertiveness was positively related with both types of workplace bullying (task-and person-related), while it affects affective commitment only through the mediation of bullying (there is no direct effect). Specific items of the assertiveness scale asked whether in the organization people tend to be (a) aggressive or non-aggressive, (b) assertive or non-assertive, (c) dominant or non-dominant and (d) tough or tender. Possibly, in organizations where people tend to be aggressive, dominant and tough, and where assertive behaviors are the norm, both person-related and task-related bullying May be considered as normal. Also, bullying May be mistaken for assertiveness, therefore no action is taken to minimize or avoid it.

The performance orientation cultural dimension on the other hand, i.e., the degree to which an organization encourages and rewards group members for performance improvement and excellence, a generally positively perceived cultural aspect that has been repeatedly linked with high performance in organizations (Che-Ha et al., 2014), consistently linked positively with work-related bullying. Ingroup collectivism in contrast, i.e., the emphasis on family/internal group bonds within an organization, consistently seems to act as a barrier to work-related workplace bullying. These findings are particularly meaningful and practical, as they suggest that companies with specific cultural profiles May be more susceptible to bullying phenomena (Pilch and Turska, 2015).

This research has also delved into the relationship between workplace bullying and organizational commitment. The occurrence of work-related workplace bullying appears to be negatively related to affective commitment, while person-related bullying does not exhibit any consistent relationship with affective commitment. This is a sensible outcome, since work-related bullying May be more easily attributed to organizational factors than person-related bullying which May be attributed more easily to specific individuals or groups, but not to the organization (Bari et al., 2023; Vandevelde et al., 2020). More specifically, work-related bullying directly undermines an employee’s ability to perform their job tasks, which can be seen as a lack of support from the organization itself (Pilch and Turska, 2015; Trépanier et al., 2013). This erodes employees’ sense of commitment and connection to the organization. Conversely, person-related bullying, which relates to employees’ reputation and social status (Trépanier et al., 2013), is often attributed to individual conflicts rather than systemic issues, so employees May not hold the organization responsible. Therefore, person-related bullying does not link with affective commitment.

In addition to addressing the original research questions, the analysis revealed that respondents’ levels of organizational commitment are influenced by the culture in their organization. Specifically, a high ingroup collectivism culture, not only prevents the occurrence of workplace bullying but also links consistently and positively with high affective commitment of individual employees.

Finally, the study identifies key differences in the effects of culture examined across several dimensions between the two survey rounds. For example, power-distance had a significant link with person-related bullying in 2012, whereas this link was not observed in 2017. This implies that power distance, enhanced the probability of person-related bullying only during boundary context conditions, i.e., during the lowest level of the Greek recession (2012), but this link was not evident during the more positive condition of economic development in 2017. This agrees with findings on lower negotiation power of weaker parties when the macro environment becomes less favorable, and to unequal sharing of deteriorations between members of organizations, with less privileged members bearing a relatively heavier burden than privileged members (Galanaki, 2020a,b). In the same vein, uncertainty avoidance showed a negative association with work-related workplace bullying in 2017 (*b* = −0.13, *p* < 0.05), contrasting with its non-significant impact in 2012.

Additionally, dimensions such as uncertainty avoidance (*b* = 0.12, *p* < 0.05), future orientation (*b* = 0.17, *p* < 0.05), and gender egalitarianism (*b* = 0.11, *p* < 0.05) were found to link positively with affective commitment in 2012, but not in 2017. This hints that cultural aspects that tend to diminish uncertainty and injustice are perceived as more important and have a positive effect on positive employee attitudes, during times of crisis (in this case 2012, deepest economic recession), but not during more stable conditions (2017-economic development).

### 5.1 Implications

Bullying was found to exhibit strong correlations with specific dimensions of organizational culture, highlighting from a different angle the necessity for organizations to actively monitor their culture. It is crucial to emphasize that organizations, accountable for ensuring a safe working environment, have an interest in reducing bullying incidents (Pilch and Turska, 2015; Thompson and Catley, 2021). This imperative does not simply arise from a sense of social responsibility and ethical obligation on the part of employers. It is also a pragmatic and realistic necessity, as it creates the conditions for fostering performance and excellence.

The results reveal that assertiveness and performance orientation cultures exhibit positive correlations with workplace bullying. Conversely, ingroup collectivism not only negatively relates to workplace bullying, but also positively relates to affective organizational commitment. Therefore, organizations fostering aggressive behaviors and prioritizing performance over all other concerns, are more prone to nurturing and encouraging bullying incidents. This agrees with a classic theme in management literature, the proposition that effective leaders are those who value both performance/ outcomes and their people/ staff/ teams. Seminal works, classic in both academia and practice have highlighted that a good balance between concern for people and concern for results is necessary for success of any leader (Blake et al., 1961). The assurance that higher management could intervene, especially in firms with high assertiveness, provides comfort, particularly when coupled with zero-tolerance practices towards bullying, introduced and implemented by the HRM department with the support of top management. These findings underscore the importance of proactive measures to foster a culture of respect and inclusivity, encouraging organizations to prioritize the monitoring and cultivation of their cultural dynamics (Kaaria and Karemu, 2024; Thompson and Catley, 2021).

On the other hand, performance orientation is strongly linked with results-oriented organizations which place ahead and reward good performance. In such environments, separating bullying behaviors from excellence and refusing to tolerate them is crucial, as they can lead to poor performance and diminish employees’ willingness to remain in the organization, particularly for those with readily available job opportunities elsewhere. In general, performance appraisal should place emphasis both on immediate work outcomes and collaboration, coupling individual performance with team-based results. Further, along with results-based orientation, human-centered sensitivity training is advised to organizations valuing performance and outcomes.

Ingroup collectivism emerges as the organizational cultural dimension that prevents bullying incidents, fostering a friendly work environment where employees support and collaborate with each other. In such environments, victims of bullying would easily find allies to support their case and incidents of bullying behaviors would be criticized and often penalized. In light of these insights, organizations are encouraged to reevaluate their cultural values and practices, seeking to cultivate environments that prioritize collaboration, trust, respect, and mutual support.

Generally, creating a zero-tolerance environment for bullying should be a top priority for HRM. Implementing anti-bullying measures is a crucial action for organizations, as previous literature provides mixed findings on the effectiveness of such programs (Salin et al., 2020). For example, studies in the context of different workplaces, such as schools (Li and Hesketh, 2021), or organizations in the transportation sector (Einarsen et al., 2018), suggest that multi-faceted intervention programs might be effective towards bullying prevention. On the other hand, the absence of a clear policy and the lack of clear communication to organizational members might give a wrongful signal to bullies that bullying is accepted as a part of organizational culture (Ahmed and Omran, 2020). Therefore, establishing channels for reporting incidents, offering sensitivity training, implementing codes of conduct or codes of ethics, and providing psychological support to affected employees are critical steps. Monitoring good human relations at the workplace and fair treatment aligns with the HRM department’s responsibility to ensure employee well-being and performance.

Regarding the relationship between workplace bullying and overall organizational commitment, the results indicate a negative association, particularly with work-related bullying and affective commitment. It is reasonable to assume that in workplaces where bullying occurs, organizational commitment tends to be lower. Therefore, individuals who have experienced work-related bullying are likely to exhibit lower levels of affective commitment to their organization, which has proven incapable of protecting them from a highly negative experience.

Finally, within organizational contexts, bullying undermines trust and cooperation among team members, ultimately impeding productivity and suffocating innovation. Furthermore, it inflicts long-term emotional and psychological trauma for victims, jeopardizing their mental health and hindering their social integration. On a larger scale, a culture that tolerates bullying sustain cycles of aggression, posing a threat to the harmony and inclusivity of communities and undermining overall social cohesion and well-being.

### 5.2 Limitations and future research

This exploratory study has common limitations inherent in its nature. It primarily identifies relationships without establishing causality. Additionally, focusing the analysis on individual employees limits the depth of insights. For more robust and meaningful results, future research should analyze data at the organizational level with larger participant groups from each organization, enabling comparisons across different organizational contexts.

Furthermore, since the research was conducted within a single country, the findings May not be generalizable to other countries. Future research should involve more extensive and large-scale investigations that compare organizations with different cultures. Conducting such research at the international level would enhance the understanding of how national culture influences organizational culture. This is valuable for multinational organizations, where discrepancies between societal and organizational cultures May be pronounced due to the diverse national backgrounds of employees. Overall, cross-cultural research can deepen our understanding of the complex interplay between organizational culture, workplace bullying, and affective commitment, providing valuable insights for organizations operating in multicultural environments and globalized markets.

Given the variability in the significance of cultural dimensions concerning workplace bullying between different research rounds, future research could explore contextual factors that May influence this impact over time, beyond conducting comparative studies across different countries and industries. Factors such as shifts in organizational policies, evolving leadership styles, economic conditions, and societal norms May play a pivotal role in shaping these dynamics.

## 6 Conclusion

Bullying carries profound social implications that extend beyond individual experiences, impacting both organizational dynamics and broader societal well-being (Pilch and Turska, 2015). The present study underscores the significant interplay between organizational culture, workplace bullying, and affective commitment. It identifies specific cultural dimensions that either exacerbate or mitigate bullying. Both work-related and, to a lesser extent, person-related bullying negatively impact affective commitment, diminishing employees’ emotional attachment to their organization. These findings highlight the necessity of cultivating positive cultural environments that discourage bullying and promote inclusivity. The study provides a foundation for future research and practical interventions aimed at enhancing organizational well-being and effectiveness.

## Data Availability

The raw data supporting the conclusions of this article will be made available by the authors, without undue reservation.

## Footnotes

^1^The GLOBE research methodology and all research instruments are publicly available at the GLOBE project website, https://www.globeproject.com/. More specifically, all research instruments, scale information, psychometric characteristics and research directions can be found on the Globe project website under Instruments Phase 2 and at https://www.globeproject.com/data/GLOBE-Dimensions-Definitions-and-Scale-Items.pdf (Globe Project, 2006).

^2^Specifically, items “Someone withholding information which affects your performance,” “Being ordered to do work below your level of competence,” “Having your opinions ignored,” and “Excessive monitoring of your work” from the Work-related bullying dimension and items ““Having key areas of responsibility removed or replaced with more trivial or unpleasant tasks,” “Spreading of gossip and rumors about you,” “Being ignored or excluded,” “Repeated reminders of your errors or mistakes” and “Persistent criticism of your errors or mistakes” from the Person-related bullying dimension of the Einarsen et al. (2009) were excluded from the analysis due to very high cross-loadings.
